# Genetic diversity and core collection of Polygonati Rhizoma in China via SSR markers

**DOI:** 10.3389/fpls.2025.1674396

**Published:** 2025-11-06

**Authors:** MingYu Zhu, XinChang Zhu, YuLing Zeng, Wei Li, Xuan Wen, Yang Mei, ChuTong Huang, ZhiGang Hu, Lin Sen

**Affiliations:** 1 College of Pharmacy, Hubei University of Chinese Medicine, Wuhan, China; 2 Traditional Chinese Medicine of Wuxue City, Wuxue, China; 3 Hubei Shizhen Laboratory, Wuhan, China

**Keywords:** Polygonati Rhizoma, SSR fluorescent markers, genetic diversity, core collection, SSR markers

## Abstract

**Introduction:**

Polygonati Rhizoma is a Traditional Chinese medicine derived from three species of the *Polygonatum* Mill. (*Polygonatum kingianum, Polygonatum sibiricum*, and *Polygonatum cyrtonema*), renowned for its culinary and medicinal uses. Despite extensive research, comprehensive studies on the population genetic diversity and core collection construction of medicinal Polygonatum species remain scarce.

**Methods:**

To address this, we employed 18 highly polymorphic SSR markers to develop two machine learning models for species discrimination. Subsequently, we performed comprehensive population genetic analyses on 175 accessions, followed by core germplasm construction.

**Results:**

The study demonstrated that the machine learning-based approach achieved consistently high discrimination accuracy, exceeding 81% for *Polygonatum* species identification. Among the four investigated Polygonati Rhizoma, significant variations in genetic diversity were observed. Cluster and population structure analyses identified three primary subgroups. A core collection was constructed through stepwise clustering based on genetic distance. The C78 primary core collection achieved an allele retention rate of 84.59%, with minimal genetic redundancy.

**Discussion:**

These findings provide a robust foundation for the conservation of medicinal Polygonatum spp. germplasm and offer potential resources for future genetic improvement and variety selection.

## Introduction

Polygonati Rhizoma, a perennial herb belonging to the genus *Polygonum* within the Liliaceae family, is recognized in the 2025 edition of the *Chinese Pharmacopoeia* as a medicinal herb derived from the dried rhizomes of three species: *Polygonatum kingianum* Coll. et Hemsl., *Polygonatum sibiricum* Red., and *Polygonatum cyrtonema* Hua (Pharmacopoeia Committee, 2025). Included in the Ministry of Health's “List of Items that are both Food and Drugs” since 2002, *Polygonati Rhizoma* stands out for its significant medicinal and nutritional value, and renowned for its nutritional and therapeutic properties (Zhang et al., 2022), it was ranked among the “Top Ten Chuyao (Hubei Provincial Featured Chinese Medicines)” in year 2022 (https://www.hubei.gov.cn/zwgk/hbyw/hbywqb/202207/t20220716_4223149.shtml). Its therapeutic functions, including tonifying Qi (vital energy—the fundamental life force in TCM) and nourishing Yin (corporeal cooling and moistening functions), moisturizing the lungs, and strengthening the spleen, are attributed to its rich chemical composition, which primarily includes polysaccharides, steroids, anthraquinones, alkaloids, lignin, vitamins, and amino acids (Ma et al., 2022; Chen et al., 2015). Extensive pharmacological research has demonstrated its diverse biological activities, including anti-aging, hypoglycemia, immune enhancement, anti-atherosclerosis, cardiovascular protection, bacteriostasis, and intestinal flora regulation (Zhou et al., 2021; Wang et al., 2019). *P. zanlanscianense* (*Polygonatum zanlanscianense* Pamp.), a closely related species to *Polygonati Rhizoma*, also exhibits both medicinal and nutritional values and is often used in traditional Chinese medicine, sometimes serving as a substitute or being confused with *Polygonati Rhizoma* in certain regions (Li et al., 2024; Song et al., 2022). *Polygonati Rhizoma* thrives in shaded habitats such as forests and sloped terrains at altitudes of approximately 1000 meters. China boasts a vast array of germplasm resources within the *Polygonatum* Mill. genus, predominantly occurring in wild environments (Cheng et al., 2020). However, the increasing research focus on traditional Chinese herbal medicine has led to overexploitation, resulting in a significant depletion of *Polygonati Rhizoma* resources in primary production areas and substantial habitat destruction. Additionally, the phenomenon of substituting other *Polygonatum* Mill. Species for traditional medicinal *Polygonati Rhizoma* has emerged. This issue stems from the high degree of similarity in both geographical distribution and morphological characteristics among species within the *Polygonatum* Mill., which has significantly undermined the sustainable development of the *Polygonati Rhizoma* industry. Therefore, it is imperative to conduct research on resource collection and conservation, develop artificial cultivation techniques, and assess genetic diversity. The establishment of a core collection bank for medicinal *Polygonati Rhizoma* will not only provide innovative solutions to address resource scarcity but also serve as a valuable reference for selection, breeding, and the establishment of new germplasm.

Simple Sequence Repeat (SSR) molecular marker technology exhibits independence from organism developmental stages, growth environments, and inter-organ/tissue variations—representing a critical advantage over phenotype-based identification approaches (such as traits assessed through leaf morphology or fruit configuration) and other externally influenced molecular markers (Liu et al., 2020). Practically, SSR markers have found widespread application across diverse research fields: genetic diversity studies in plant germplasm resources (Liu et al., 2020; Yang et al., 2024), investigations into species evolutionary lineages (Thakur et al., 2017), establishment of DNA fingerprinting systems for germplasm characterization (Wu et al., 2024), and construction of genetic linkage maps (Zheng et al., 2023). Within the genus *Polygonatum* Mill. (valuable medicinal plants with dual economic and therapeutic significance), multiple polymorphic SSR loci have been characterized. Nevertheless, prior investigations predominantly focus on single species including *P. cyrtonema* (Cheng et al., 2020; Liu et al., 2023; Ji et al., 2020; Chen et al., 2024; Cheng, 2023), *P. kingianum* (Mi et al., 2024; Qian et al., 2023; Chen et al., 2022), and *P. sibiricum* (Yang et al., 2022). Crucially, a notable research void exists regarding comprehensive studies on the three pharmacopoeia-designated species of *Polygonati Rhizoma*, with existing works suffering from inadequate sample sizes (Yang et al., 2022; Feng et al., 2020; Zhu et al., 2018; Wang et al., 2018).

The global consensus underscores the critical role of germplasm resource collection and conservation in facilitating the selection and breeding of novel plant varieties, as well as in enhancing specific germplasm materials. As these resources accumulate, there is a significant rise in management costs and challenges associated with selecting and mining specific germplasm. Core germplasm collections are vital as they represent the entire genetic diversity of all resources with minimal resources and genetic duplications (Frankel and Brown, 1984; Frankel, 1984). Consequently, developing core germplasm collections is a strategic approach for comprehensive evaluation, efficient conservation, and optimal utilization of germplasm resources. The construction of core collections aims to preserve maximum genetic variation using minimal resources, marking it as a crucial area of research in plant germplasm management (Koorevaar et al., 2024). Given the detailed investigation into the current status of *Polygonati Rhizoma* germplasm resources, constructing a core collection is essential for improving the efficiency of management, conservation, evaluation, innovation, and utilization of *Polygonati Rhizoma* germplasm resources.

In this study, a total of 175 rhizome accessions from *P. cyrtonema*, *P. kingianum*, *P. sibiricum*, and *P. zanlanscianense* were collected across various geographical regions. Using SSR markers, the research systematically analyzed the kinship, genetic diversity, and population structure of the *Polygonati Rhizoma* germplasm to construct a core collection. This work provides crucial technical support for future variety selection and molecular identification of interspecific species within the genus *Polygonatum*.

## Material and methods

### Plant materials

To guarantee the rationality and representativeness of population delimitation during sampling, two accessions of Polygonati Rhizoma were considered to belong to the same population when their absolute geographical distance was less than 5 km. A total of 175 accessions of Polygonati Rhizoma were collected from 35 locations across 13 provinces in China (**Table 1**), encompassing four species: *P. kingianum* (41), *P. cyrtonema* (76), *P. sibiricum* (54), and *P. zanlanscianense* (4). All plant materials were identified by Associate Professor Lin Sen at Hubei University of Chinese Medicine. The collection was conducted with proper authorization and in accordance with the regulations of Hubei University of Chinese Medicine and national guidelines. The specimens are preserved in the Herbarium of Hubei University of Chinese Medicine (Associate Professor Lin Sen: 239011250@qq.com) and have been transplanted to the Tongxin Lake Resource Nursery of Hubei University of Chinese Medicine (N30°26′50.91″, E114°15′33.93″, 14m, Hubei, China). The specimens voucher number:HBZYY-HJZYP-0001~HBZYY-HJZYP-0175 (**Supplementary Table S1**).

**Table 1:** Information table of Polygonati Rhizoma sample collection.

No.	Population / sample name	Germplasm name	Geographical origin	Quantities	Longitude	Latitude
1	DHJ/GXD1	*Polygonatum kingianum* Coll. et Hemsl.	Baise, Guangxi	7	104.952	24.618
2	DHJ/YND2	*Polygonatum kingianum* Coll. et Hemsl.	Tengchong, Yunnan	9	98.515	25.418
3	DHJ/YND4	*Polygonatum kingianum* Coll. et Hemsl.	Chuxiong, Yunnan	8	102.713	23.769
4	DHJ/YND3	*Polygonatum kingianum* Coll. et Hemsl.	Jianshui, Yunnan	10	101.248	24.453
5	DHJ/YND5	*Polygonatum kingianum* Coll. et Hemsl.	Chuxiong, Yunnan	7	101.303	24.655
6	DH/AHDH2	*Polygonatum cyrtonema* Hua	Lu'an, Anhui	4	115.544	31.248
7	DH/AHDH3	*Polygonatum cyrtonema* Hua	Xuancheng, Anhui	6	119.022	30.393
8	DH/AHDH4	*Polygonatum cyrtonema* Hua	Chizhou, Anhui	4	117.757	30.508
9	DH/CQDH5	*Polygonatum cyrtonema* Hua	Qianjiang, Chongqing	4	108.689	29.616
10	DH/HBDH6	*Polygonatum cyrtonema* Hua	Xianning, Hubei	4	114.111	29.419
11	DH/HBDH11	*Polygonatum cyrtonema* Hua	Xianning, Hubei	2	114.130	29.487
12	DH/GZDH13	*Polygonatum cyrtonema* Hua	Qiandongnan, Guizhou	7	109.228	26.714
13	DH/HNDH15	*Polygonatum cyrtonema* Hua	Xiangxi, Hunan	5	109.506	28.027
14	DH/HNDH17	*Polygonatum cyrtonema* Hua	Yongzhou, Hunan	3	111.913	26.661
15	DH/JXDH20	*Polygonatum cyrtonema* Hua	Jiujiang, Jiangxi	1	114.499	28.921
16	DH/HBDH27	*Polygonatum cyrtonema* Hua	Yichang, Hubei	4	110.522	30.251
17	DH/HBDH28	*Polygonatum cyrtonema* Hua	Xiangyang, Hubei	1	111.666	32.222
18	DH/ZJDH29	*Polygonatum cyrtonema* Hua	Hangzhou, Zhejiang	4	119.119	29.849
19	DH/HBDH21	*Polygonatum cyrtonema* Hua	Shiyan, Hubei	4	110.350	31.814
20	DH/HNDH30	*Polygonatum cyrtonema* Hua	Luoyang, Henan	8	112.122	33.792
21	DH/SXDH31	*Polygonatum cyrtonema* Hua	Shangluo, Shaanxi	3	110.890	33.524
22	DH/HBDH22	*Polygonatum cyrtonema* Hua	Xiangyang, Hubei	6	111.666	32.222
23	DH/HBDH25	*Polygonatum cyrtonema* Hua	Xiangyang, Hubei	3	111.318	32.099
24	DH/XZDH32	*Polygonatum cyrtonema* Hua	Rikaze, Tibet	3	85.283	28.414
25	HB/HBHB	*Polygonatum zanlanscianense* Pamp.	Yichang, Hubei	4	110.522	30.295
26	HJ/SCHJD	*Polygonatum sibiricum* Red.	Suining, Sichuan	3	105.517	30.558
27	HJ/SCHJE	*Polygonatum sibiricum* Red.	Nanchong, Sichuan	9	106.530	30.797
28	HJ/SXHJH	*Polygonatum sibiricum* Red.	Hanzhong, Shaanxi	4	107.541	33.220
29	HJ/CQHJA	*Polygonatum sibiricum* Red.	Liangping, Chongqing	8	107.568	30.547
30	HJ/HBHJB	*Polygonatum sibiricum* Red.	Xianning, Hubei	1	114.130	29.487
31	HJ/GZHJC	*Polygonatum sibiricum* Red.	Yanhe, Guizhou	8	108.503	28.564
32	HJ/HNHJG	*Polygonatum sibiricum* Red.	Xiangxi, Hunan	8	109.506	28.027
33	HJ/SCHJJ	*Polygonatum sibiricum* Red.	Ziyang, Sichuan	3	105.560	30.067
34	HJ/SCHJK	*Polygonatum sibiricum* Red.	Suining, Sichuan	4	105.517	30.558
35	HJ/HBHJP	*Polygonatum sibiricum* Red.	Yichang, Hubei	6	110.522	30.251

Rhizome dehydration was performed as a routine but critical step to extend sample shelf-life and prevent DNA degradation during post-sampling transport and storage (a standard practice in plant molecular studies for non-freshly analyzed materials). Specifically, rhizomes were dehydrated in a forced-air oven at 45°C for 48 h until constant weight was achieved—this temperature and duration balance efficiency and DNA integrity, avoiding thermal damage to genomic DNA.

### DNA extraction

To ensure genomic DNA integrity during sample pretreatment and facilitate subsequent extraction, the rhizomes of Polygonati Rhizoma were first dehydrated using silica gel—this desiccation method effectively absorbs moisture from the rhizome tissue, preventing microbial growth and DNA degradation while maintaining tissue structure for downstream processing. After dehydration, the rhizome samples were pulverized to a fine powder using a mortar and pestle under liquid nitrogen (to avoid DNA shearing from heat generated during grinding).

Genomic DNA extraction was then performed strictly according to the manufacturer's instructions provided with the NuClean Plant Genomic DNA Kit. Following extraction, the concentration and purity of the obtained genomic DNA were quantified using a NanoDrop 2000 Ultra-Micro Spectrophotometer (assessing A260/A280 ratios to ensure purity, with optimal values ranging from 1.8 to 2.0). DNA quality was further verified by 1%(W/V) agarose gel electrophoresis, where clear, intact bands (without smearing) indicated high-quality genomic DNA.

To standardize samples for subsequent experiments (e.g., SSR amplification), the DNA concentration of all samples was adjusted to 50 ng/µL using TE buffer. The final standardized DNA samples were stored at -20 °C to maintain stability, ensuring they were suitable for subsequent molecular experiments.

### Primer screening and SSR-PCR amplification

Based on literature-reported polymorphism and primer amplification (Cheng et al., 2020; Liu et al., 2023; Cheng, 2023; Qian et al., 2023; Chen et al., 2022; Yang et al., 2022; Feng et al., 2020; Wang et al., 2018; Cheng, 2012), a total of 120 pairs of fluorescent primers were selected and analyzed in this study (**Supplementary Table S2**). Primers were synthesized by Bioengineering (Shanghai) Co. The PCR reaction system comprised a total volume of 20 µL, including 3 µL genomic DNA, 10 µL 2× Es Taq MasterMix, 1.5 µL forward primer, 1.5 µL reverse primer, 0.3 µL M13 connector (ROX/HEX/FAM), and 4 µL ddH_2_O. The PCR amplification conditions included pre-denaturation at 94°C for 2 minutes, followed by 35 cycles of denaturation at 94°C for 30 seconds, annealing at the optimal temperature for 30 seconds (note that the annealing temperature varied according to the specific SSR primers used), extension at 72°C for 30 seconds, with a final extension at 72 °C for 2 minutes, and storage at 4°C. The PCR amplified products were analyzed using agarose gel electrophoresis (1%(W/V)), followed by fluorescence capillary electrophoresis.

The raw data were analyzed using GeneMarker v2.2.0 software (Holland et al., 2017). Primers exhibiting distinct peak shapes, smooth baselines, and significant polymorphisms were selected for large-scale amplification. Ultimately, 18 highly polymorphic SSR primer pairs were chosen for further analysis (**Table 2**).

**Table 2:** Detailed information on 18 pairs of polymorphic SSR primers used for genetic diversity analysis and core collection.

Primer no.	Primer sequences	Repeat motifs	Optimal annealing temperature/°C
S3	F: TGGTTTGTCACCCTGCAAGA R: AAGCAAACCGTACTGTTGCG	(AAAAG) 5	53
S4	F: ACCCATACTCGTGTCTGCTC R: AGCATGCAGGAGAGTGAAGA	(AAAAG) 5	53
S13	F: CCGCTCTCTTTCCCTCTCTT R: TCGTCGGAGCTAGTGAATCC	(AAG) 8	53.5
S15	F: GGTGAGATTTGGCGAAGAAC R: AGGGATCCACTGCTCCTCTT	(AAT) 5	52
S21	F: TCTTGCTCTACCTCCTTGCTTCT R: GTCACACCTTTCCCTCTACTTAAC	(AG) 48	52.5
S27	F: CCAACAAGGGAAAAGCAAAG R: AATATGGGGGTGACTCCGTT	(AGAA) 6	51
S34	F: GGACACAAACACAGCGACAC R: TGTTTTCAAATCTTTTGGAGTCTGA	(AT) 7	50
S52	F: TGACCCTCCACTTCATCTCC R: GACTCCTTCGAGTGGTACGC	(CGG) 8	53
S55	F: TCTGTAAATCAGGAGGAGCGA R: AATCGATGAGATCGACCTGG	(CT) 12	52
S69	F: AGCAAGAAAAGGGCACTCG R: CCCTCCCCTTCATAATTGCT	(GAA) 3 (AG) 34	51.5
S83	F: CCGATGACAGGATCGACGTT R: TCCTCACCCACAACACCAAC	(GGA) 5	55
S96	F: TCAAGGAGACGAGGGAGAGG R: CTGAAAAGCCAGATGCAGAGC	(TC) 7gctctctctct (CA) 7	55.5
S99	F: CCTCCGCTACCACTTCCATT R: ACCCCAATCCCTAACCCTGA	(TCG) 5	55.5
S101	F: GTCCGAGTTCTTTGACGAGC R: AAAACCATCTCCATCCTCCC	(TG) 10 (AG) 6	52
S102	F: GAGGTGATCTCGGCATCCAG R: TACACCCTGCTCTCTCCCAA	(TG) 7	55.5
S105	F: GATTCGCTAGCGCAAAACGT R: ACCAGAACCAAGTAAGCAGCA	(TGTA) 6	53
S109	F: ACCCAACCCCAAACATCGTT R: CTGGAGATCAGAGACGACGC	(TTAA) 6	55.5
S113	F: CCCCCTTCGCTTTCTCTGTT R: GCCCAGCCCTCAAAGTTTTG	(TTCA) 5	55

### Data analysis

A presence-absence matrix, coded as ‘1’ for presence and ‘0’ for absence of specific allelic variants, was constructed based on allelic data obtained from 18 highly polymorphic SSR primer pairs (**Supplementary Table S3**). This coding scheme, which indicates the presence or absence of a particular allele at each locus, was implemented to facilitate analysis using GenAlEx v6.5 software (Peakall and Smouse, 2012). Using this software, we calculated genetic diversity parameters including allele frequencies, observed number of alleles (*Na*), effective number of alleles (*Ne*), observed heterozygosity (*Ho*), expected heterozygosity (*He*), and Shannon's information index (I). GenAlEx v6.5 was also used to perform Analysis of Molecular Variance (AMOVA) and compute genetic differentiation coefficients (*Fst*). The polymorphism information content (PIC) was determined using PowerMarker v3.25 software (Liu and Muse, 2005). For cluster analysis, Neighbor-Joining (NJ) trees were constructed with the R package ape (Paradis and Schliep, 2018), and the resulting tree files were visualized and annotated using the online tool iTOL v6.5.8 (Letunic and Bork, 2021). Population structure analysis was conducted using STRUCTURE software.

### Development of SSR-based predictive models for Polygonati Rhizoma authentication

In this study, we employed two supervised classification models (Support Vector Machine-Recursive Feature Elimination (SVM-RFE) and Random Forest (RF)) for population identification using SSR markers (Agho et al., 2024). The SVM is a supervised machine learning model that utilizes kernel methods for nonlinear classification by constructing hyperplanes for discrimination. The SVM-RFE approach, built upon the SVM framework, employs recursive feature elimination to extract significant correlated variables. This iterative process trains the model to score and rank each feature, sequentially removing the lowest-scoring features until the optimal number of features is selected. The RF model operates through an ensemble of decision trees, providing inherent resistance to overfitting (Niederer et al., 2024). For SVM-RFE implementation, we utilized the caret package in R. The training and test set ratios were determined through multiple iterations based on learning curve accuracy. Model parameters were optimized via 10-fold cross-validation of the training matrix, with validation sets used to assess predictive performance. Feature elimination was performed using the e1071 package. The RF model was constructed using R's randomForest and rfPermute packages, with the number of decision trees ultimately set to 300. Model performance was rigorously evaluated through confusion matrices, accuracy rates, precision, and F1 scores to ensure reliability. Due to limited sample availability for *P. zanlanscianense*, the final dataset comprised SSR markers from *P. sibiricum*, *P. cyrtonema*, and *P. kingianum*.

### Core collection construction

To optimize the conservation of genetic diversity within the germplasm resources of Polygonati Rhizoma while minimizing the number of samples, we employed a Least Distance Stepwise Sampling (LDSS) strategy in this study. This method specifically aims to identify the group exhibiting the smallest genetic distance and the highest genetic similarity. Following the construction of the core subset, we compared the genetic diversity parameters of this core collection with those of the original collection. These comparisons were visualized through Principal Component Analysis (PCA) and Neighbor-Joining (NJ) trees. Finally, the reliability of the core collection was validated using a T-test conducted in SPSS software, comparing the core samples against the overall sample set.

## Results

### Genetic diversity analysis of SSR molecular marker primers

A total of 18 highly polymorphic SSR primer pairs were rigorously selected from an initial pool of 120 candidates (**Table 2**), with the corresponding analysis results systematically presented in **Table 3**. The fluorescent capillary electrophoresis profiles are shown in **Supplementary Figure S1**. These 18 primer pairs successfully amplified a total of 186 alleles, with an average of 15.89 alleles per locus. The calculated core genetic diversity parameters were as follows: the mean effective number of alleles (*Ne*) was 2.817, observed heterozygosity (*Ho*) was 0.016, expected heterozygosity (*He*) was 0.466, mean Shannon's information index (*I*) was 0.997, and mean polymorphic information content (PIC) was 0.775—all parameters met the criteria for highly polymorphic markers (1.0 > PIC > 0.5). Among the primers, primer S83 exhibited the highest polymorphism (PIC = 0.909, **Supplementary Figure S1**), followed by primer S99 (PIC = 0.902, **Supplementary Figure S1**). The above data indicate that the developed primers possess extremely high polymorphism, and the 175 Polygonati Rhizoma germplasm resources exhibit rich and significant genetic diversity.

**Table 3:** Genetic diversity parameters for the overall samples of Polygonati Rhizoma.

Maker no.	*N*	*Na*	*Ne*	*I*	*Ho*	*He*	*PIC*
S3	24.500	13	1.429	0.700	0.000	0.335	0.771
S4	24.000	12	2.405	0.971	0.054	0.496	0.825
S13	20.000	14	2.322	0.894	0.013	0.397	0.564
S15	24.750	10	1.583	0.657	0.000	0.351	0.745
S21	19.500	13	2.755	1.052	0.000	0.578	0.757
S27	24.500	17	2.026	0.920	0.000	0.443	0.808
S34	20.000	26	3.681	1.242	0.000	0.524	0.899
S52	26.500	30	6.040	1.534	0.006	0.589	0.889
S55	34.500	11	1.294	0.572	0.167	0.301	0.595
S69	35.750	8	2.191	0.931	0.007	0.486	0.669
S83	28.750	30	4.323	1.625	0.039	0.683	0.909
S96	14.750	8	0.974	0.320	0.000	0.153	0.641
S99	12.250	18	4.098	1.286	0.000	0.594	0.902
S101	16.500	13	1.911	0.766	0.000	0.363	0.770
S102	17.750	22	4.924	1.360	0.000	0.563	0.869
S105	16.000	10	2.623	1.088	0.000	0.589	0.764
S109	12.750	9	2.020	0.782	0.000	0.384	0.738
S113	28.000	22	4.097	1.253	0.000	0.551	0.826

**Figure 1:**
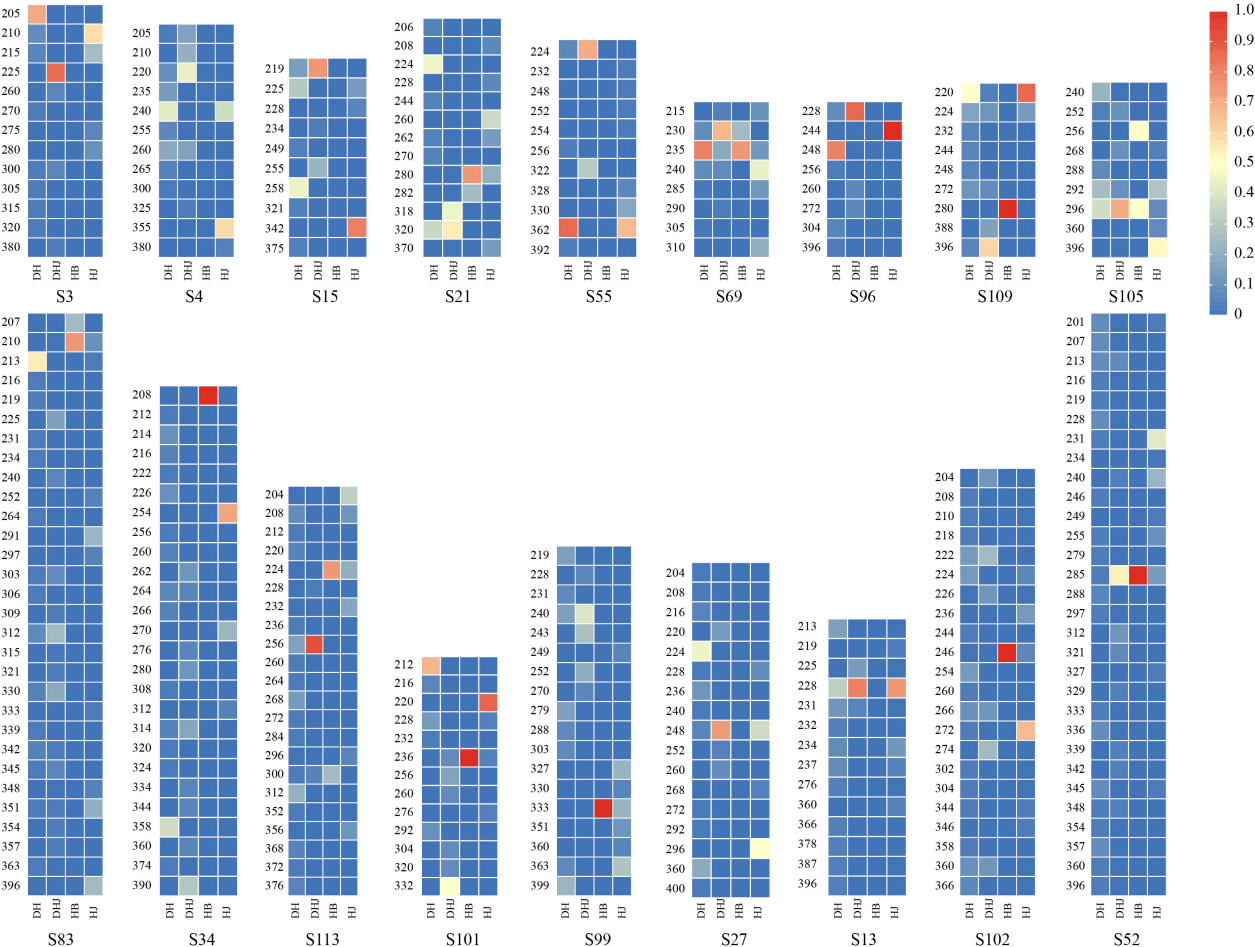
Heat map of allele frequencies of 18 pairs of primers.

Subsequently, high-frequency alleles within each species population were identified based on allele frequencies and visualized in a heatmap (**Figure 1**). Specifically, the *P. cyrtonema* population showed dominant alleles at 7 loci (S3, S55, S69, S83, S96, S101, S109); *P. kingianum* had characteristic alleles at 13 loci (S3, S13, S15, S21, S27, S52, S55, S69, S96, S101, S105, S109, S113); the *P. sibiricum* population displayed characteristic alleles at 12 loci (S3, S4, S13, S15, S27, S34, S55, S96, S101, S102, S105, S109); and *P. zanlanscianense* exhibited characteristic alleles at 11 loci (S21, S34, S52, S69, S83, S99, S101, S102, S105, S109, S113). Notably, loci such as S15, S21, S83, and S96 had preferential alleles across multiple populations, demonstrating both high genetic diversity and polymorphism.

### Species prediction of Polygonati Rhizoma based on different algorithms

#### SVM-RFE model

**Figure 2:**
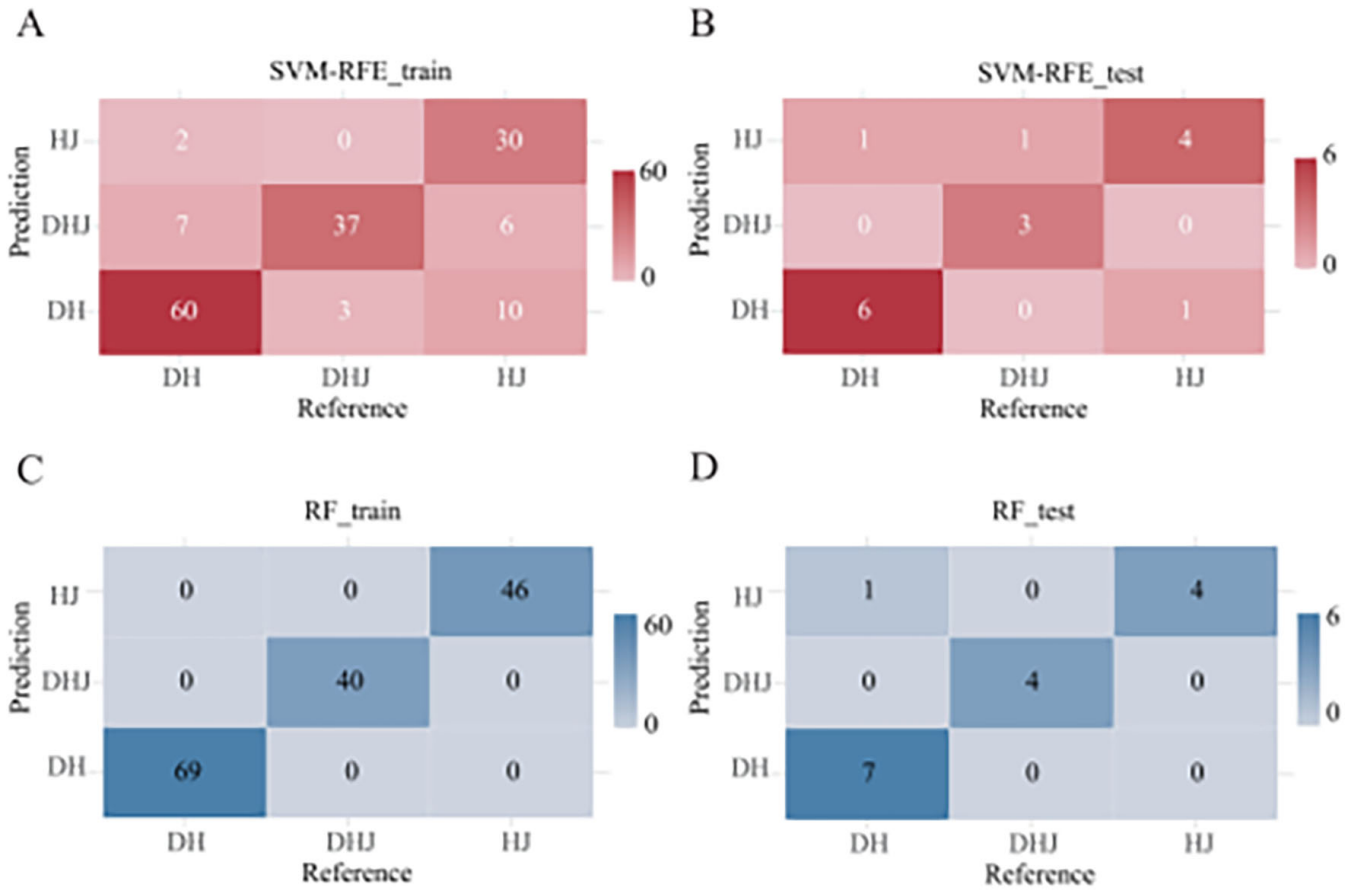
Confusion matrices. **(A)** SVM-RFE model training set; **(B)** SVM-RFE model test set; **(C)** RF model training set; **(D)** RF model test set. (The x-axis represents true species labels, while the y-axis indicates predicted species labels. Cell values denote sample counts.).

**Figure 3:**
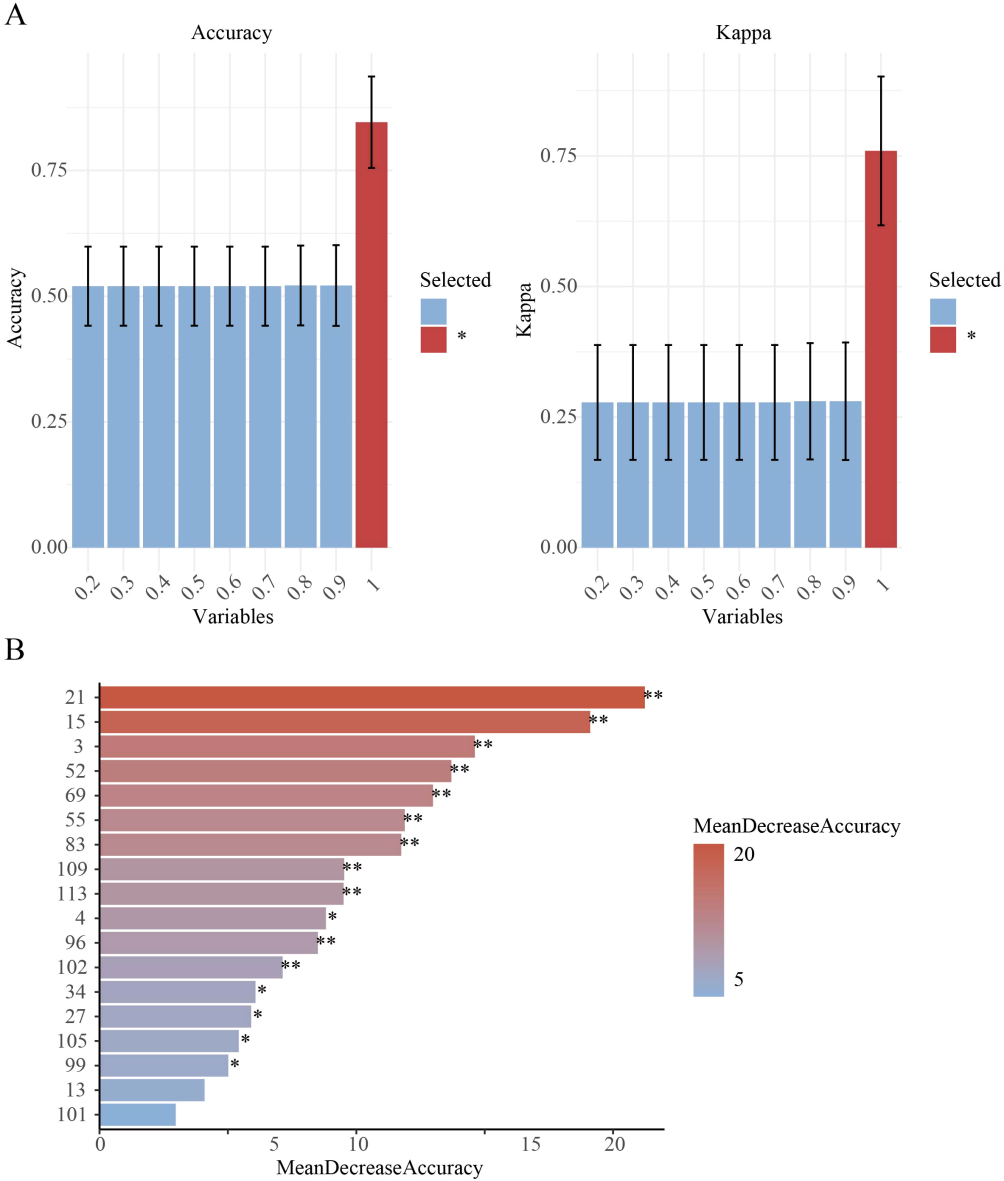
Feature importance analysis. **(A)** SVM-RFE feature selection parameters, Variables indicates the subset size/proportion of features used in training, Accuracy represents classification accuracy on the validation set (percentage of correctly predicted samples), Kappa (Cohen's Kappa coefficient) measures classification consistency, * denotes the optimal model selection; **(B)** RF feature importance: MeanDecreaseAccuracy quantifies a feature's contribution by measuring the reduction in prediction accuracy when its values are permuted (**p < 0.01, *p < 0.05).

When using the SVM-RFE model, 90% of the dataset was allocated as the training set (**Supplementary Figure S2**) and 18 primer variables were used as input. After optimization, the model performance was as follows: the classification accuracy of the training set reached 81.94% (**Figure 2A**), the classification accuracy of the validation set was 81.25% (**Figure 2B**), and the overall classification accuracy of the test set was 81.25%. Comparative analysis of precision and F1-scores revealed that the model had better classification performance for *P. cyrtonema* and *P. kingianum* than for *P. sibiricum* (**Supplementary Table S5**). Through recursive feature elimination, 5 primers with the strongest discriminative ability were selected, ranked by importance as: 34, 3, 21, 96, 109 (**Figure 3A**). Although these 5 primers could be used to construct a simplified classification model, the recursive feature selection parameters showed that the complete model with 18 primers achieved the optimal performance (**Supplementary Table S4**).

#### RF model

To enable direct performance comparison with the SVM-RFE model, the RF model used the same proportion of the training set. The results showed that the model achieved an accuracy of 100% on the training set (**Figure 2C**) and 93.75% on the validation set (**Figure 2D**). It had strong classification ability for *P. cyrtonema* and *P. kingianum*, but the recall rate for *P. sibiricum* was slightly lower, indicating potential under-prediction for this species (**Supplementary Table S5**). Feature importance analysis (**Figure 3B**) showed that the top 5 primers with the highest contribution were: 21, 15, 3, 69, 55.

### Genetic diversity analysis of Polygonati Rhizoma populations

#### Genetic diversity of populations from different species

Based on SSR amplification results, genetic diversity analysis was conducted on populations from different species (**Table 4**). Among them, the mean number of alleles (*Na*) of the DH, DHJ, HJ, and HB populations was 9.667, 5.056, 4.944, and 0.889, respectively; the mean effective number of alleles (*Ne*) was 4.869, 2.825, 2.772, and 0.800, respectively; Shannon's information index (*I*) was 1.638, 1.099, 1.089, and 0.163, respectively; the mean observed heterozygosity (*Ho*) was 0.000, 0.042, 0.021, and 0.000, respectively; and the mean expected heterozygosity (*He*) was 0.676, 0.535, 0.540, and 0.111, respectively. Among all populations, the DH population had the highest values for all genetic diversity parameters, indicating that it had the richest genetic diversity and a more extensive genetic variation pool. In addition, the observed heterozygosity (*Ho*) of all populations was lower than the expected heterozygosity (*He*), suggesting limited gene flow within these populations.

**Table 4:** Genetic diversity among the populations of four distinct species.

Species population	*Na*	*Ne*	*I*	*Ho*	*He*
DH	9.667	4.869	1.638	0.000	0.676
DHJ	5.056	2.825	1.099	0.042	0.535
HJ	4.944	2.772	1.089	0.021	0.540
HB	0.889	0.800	0.163	0.000	0.111

**Figure 4:**
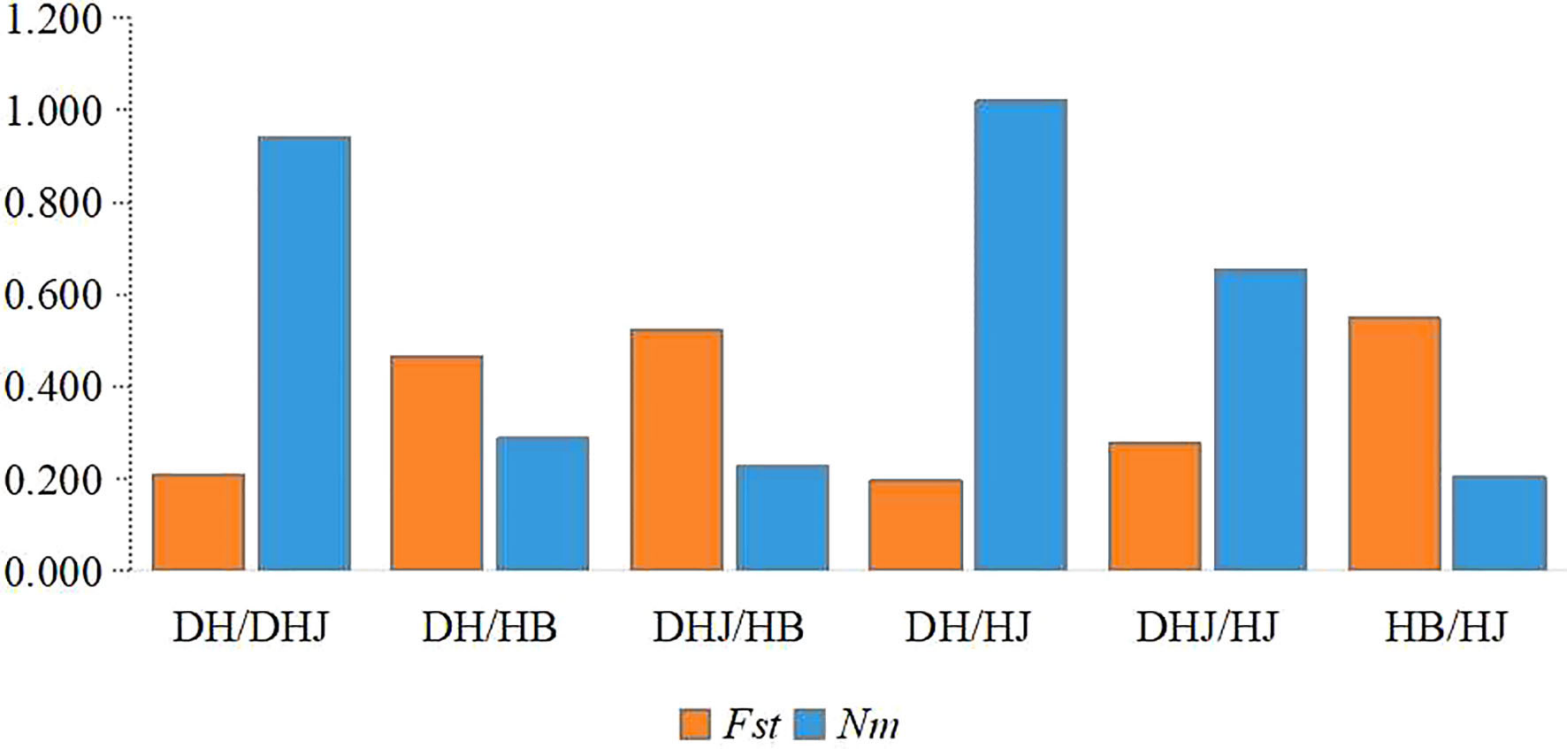
Coefficient of pairwise genetic differentiation (*Fst*) along with the level of gene flow (*Nm*) among populations.

Pairwise genetic differentiation coefficients (*Fst*) and gene flow (*Nm*) among the four populations were analyzed (**Figure 4**). The results showed significant genetic differentiation among populations, particularly between the HB and HJ populations, and between the DH and DHJ populations. In contrast, the genetic differentiation between the HJ and DH populations was relatively low (*Fst* = 0.197), accompanied by higher gene flow (*Nm* = 1.019).

#### Genetic diversity of populations from different geographic regions

Genetic diversity analysis was performed on 35 Polygonati Rhizoma populations from different geographic regions (**Table 5**). The results showed significant genetic differentiation among populations of the same species from different regions. Among the DH populations, the GZDH13 population from Qiandongnan, Guizhou Province, had the highest values for all genetic diversity parameters, which were significantly higher than those of other DH populations. Specifically, the mean number of alleles (*Na*), mean effective number of alleles (*Ne*), and Shannon's information index (*I*) of DH populations from different geographic regions ranged from 0.389 to 1.556, 0.389 to 1.470, and 0.000 to 0.353, respectively. For HJ populations, these diversity indices across different geographic populations ranged from 0.278 to 1.611 for Na, 0.278 to 1.418 for Ne, and 0.000 to 0.397 for *I*. Overall, the genetic diversity among DHJ populations showed minimal variation.

**Table 5:** Genetic diversity in 35 geographic populations.

Geographic population	*N*	*Na*	*Ne*	*I*
AHDH2	2.722	1.444	1.359	0.328
AHDH3	3.556	1.222	1.159	0.229
AHDH4	2.167	1.000	0.944	0.136
CQDH5	1.944	0.833	0.789	0.101
GZDH13	4.722	2.778	2.363	0.859
HBDH11	1.111	1.000	1.000	0.154
HBDH6	1.833	0.833	0.793	0.128
HBDH27	2.444	1.056	1.033	0.131
HBDH28	0.611	0.611	0.611	0.000
HNDH15	2.167	1.333	1.263	0.256
HNDH17	0.778	0.389	0.389	0.039
JXDH20	0.389	0.389	0.389	0.000
ZJDH29	2.667	1.167	1.111	0.175
HBDH21	2.389	1.556	1.470	0.353
HBDH22	1.611	1.000	0.959	0.189
HBDH25	0.833	0.611	0.611	0.100
HNDH30	1.278	1.111	1.083	0.263
SXDH31	0.667	0.611	0.611	0.138
XZDH32	0.889	0.556	0.556	0.116
GXD1	4.222	1.611	1.462	0.425
YND2	6.056	2.056	1.620	0.453
YND4	6.278	2.111	1.739	0.555
YND3	6.111	1.722	1.407	0.432
YND5	3.556	0.944	0.867	0.080
HBHB	1.667	0.889	0.800	0.163
SCHJD	1.611	1.278	1.233	0.241
SCHJE	2.833	1.167	0.974	0.297
SXHJH	2.889	1.167	1.144	0.131
CQHJA	5.278	1.000	0.949	0.057
GZHJC	5.333	1.611	1.418	0.397
HBHJB	0.278	0.278	0.278	0.000
HBHJP	2.611	1.278	1.146	0.287
HNHJG	3.000	0.778	0.649	0.138
SCHJJ	1.056	0.611	0.611	0.077
SCHJK	1.500	0.778	0.705	0.140
Mean	2.544	1.108	1.014	0.216

**Figure 5:**
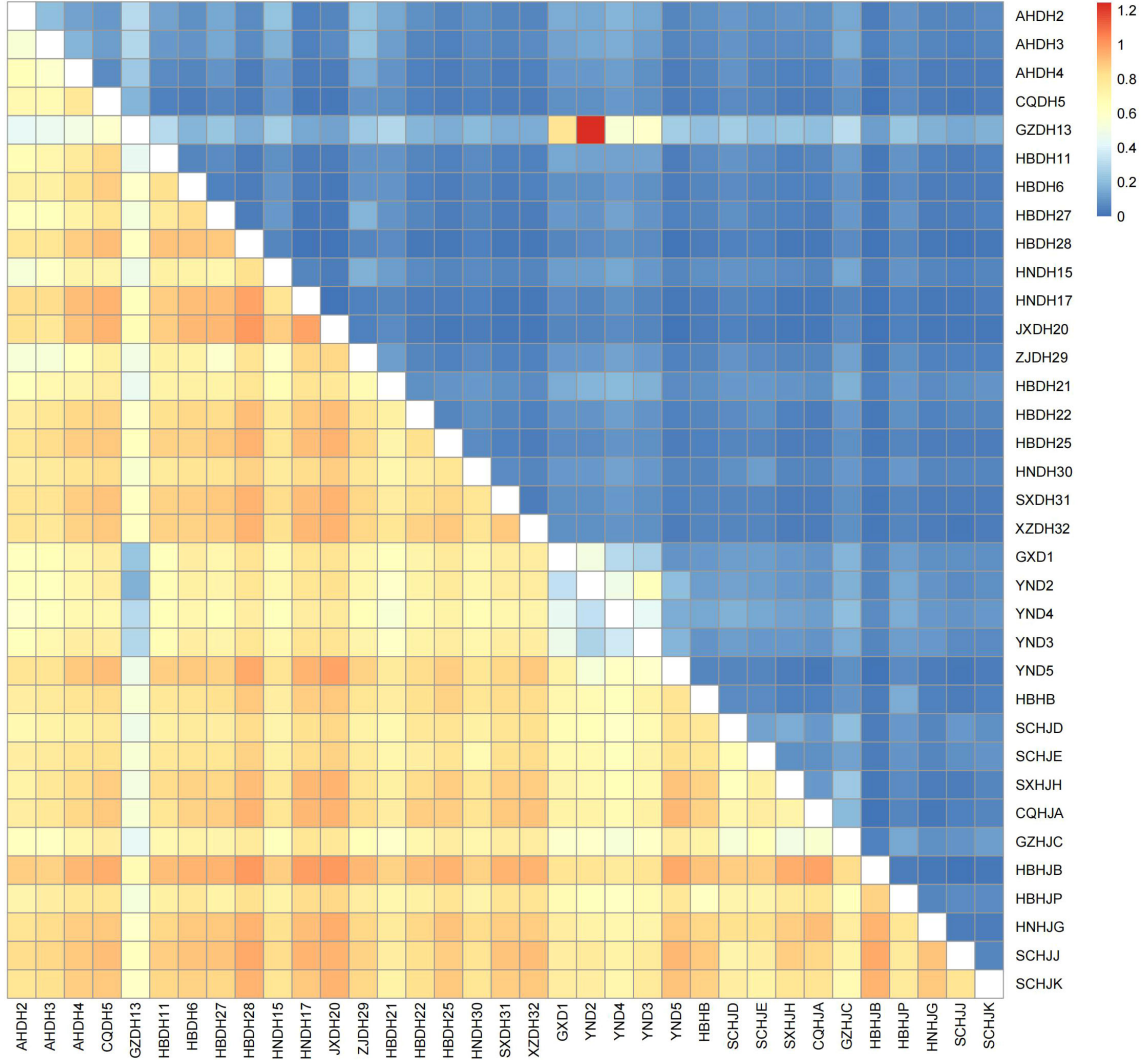
Heatmap illustrating the pairwise genetic differentiation coefficients (*Fst*) between colonies and corresponding gene flow levels (*Nm*). (Lower left quadrant: *Fst* values; Upper right quadrant: *Nm* values).

The results of pairwise genetic differentiation (*Fst*) and gene flow (*Nm*) analysis of the 35 *Polygonatum* populations are shown in **Figure 5**. Among them, the DH region populations JXDH20 and HBDH28 exhibited the highest genetic differentiation (*Fst* = 1.000), followed by the HJ region populations HBHJB and CQHJA (*Fst* = 0.970). In contrast, the genetic differentiation among populations within the DHJ group was relatively low. In addition, the GZDH13 population had a closer genetic relationship with the YND2 population (mean *Fst* = 0.301), with higher gene flow observed between these two populations (*Nm* = 1.243).

**Figure 6:**
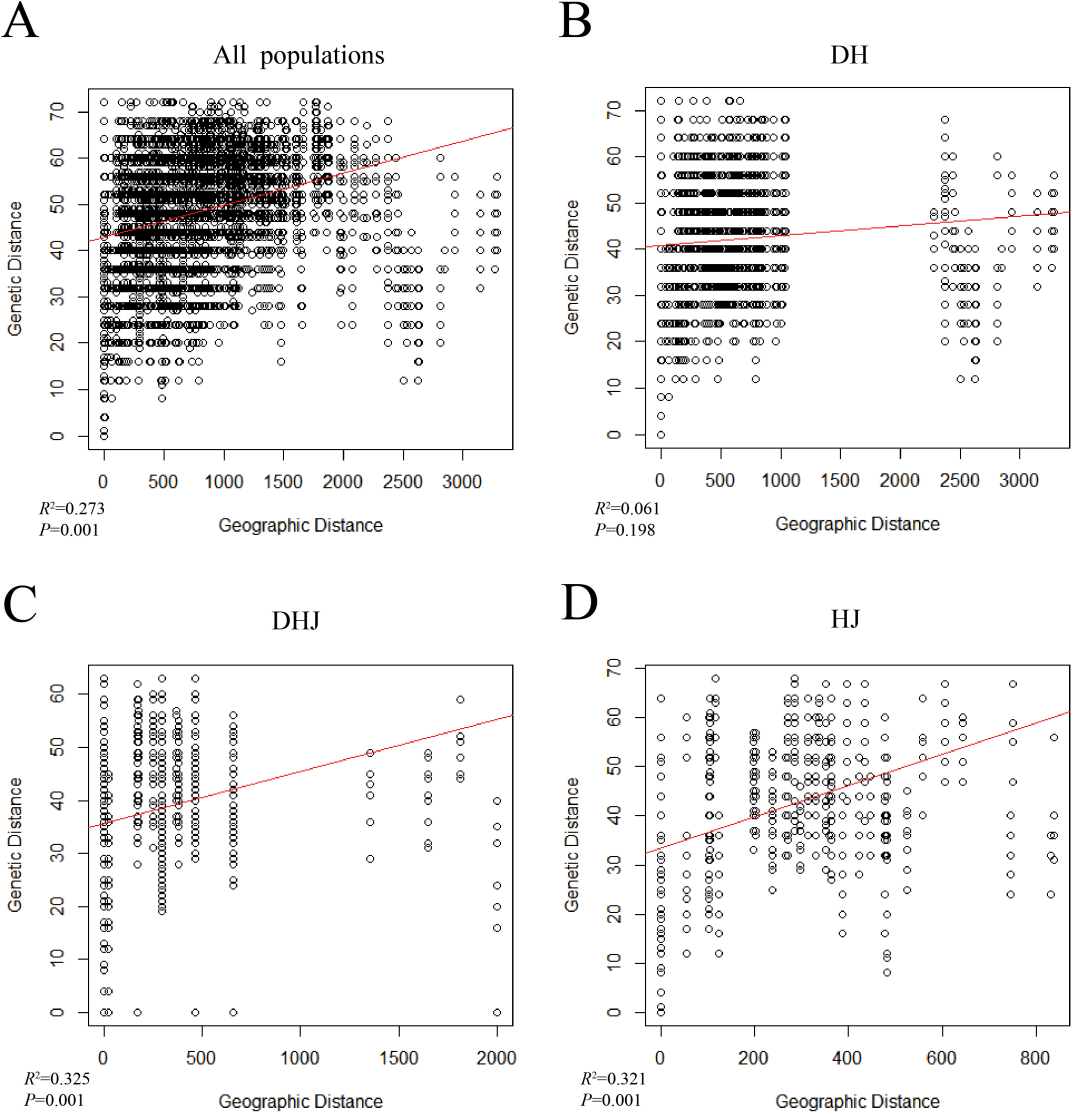
Mantel's test analysis of the populations of *Polygonatum* Mill. **(A)** all populations; **(B)***P. cyrtonema* population (DH); **(C)***P. kingianum* population (DHJ); **(D)***P. sibiricum* population (HJ).

The results of AMOVA analysis (**Supplementary Table S6**) showed that most of the genetic variation in *Polygonatum* Mill. occurred within populations, and the inter-population variation of *P. sibiricum* accounted for 54%, which was the main source of intraspecific variation. The results of the Mantel test (**Figure 6**) indicated a significant positive correlation between genetic distance and geographic distance among the 35 *Polygonatum* populations (R² = 0.273, *P* = 0.001); this significant correlation still existed when *P. cyrtonema*, *P. kingianum*, and *P. sibiricum* were analyzed individually.

### Kinship and population structure analysis of Polygonati Rhizoma populations

**Figure 7:**
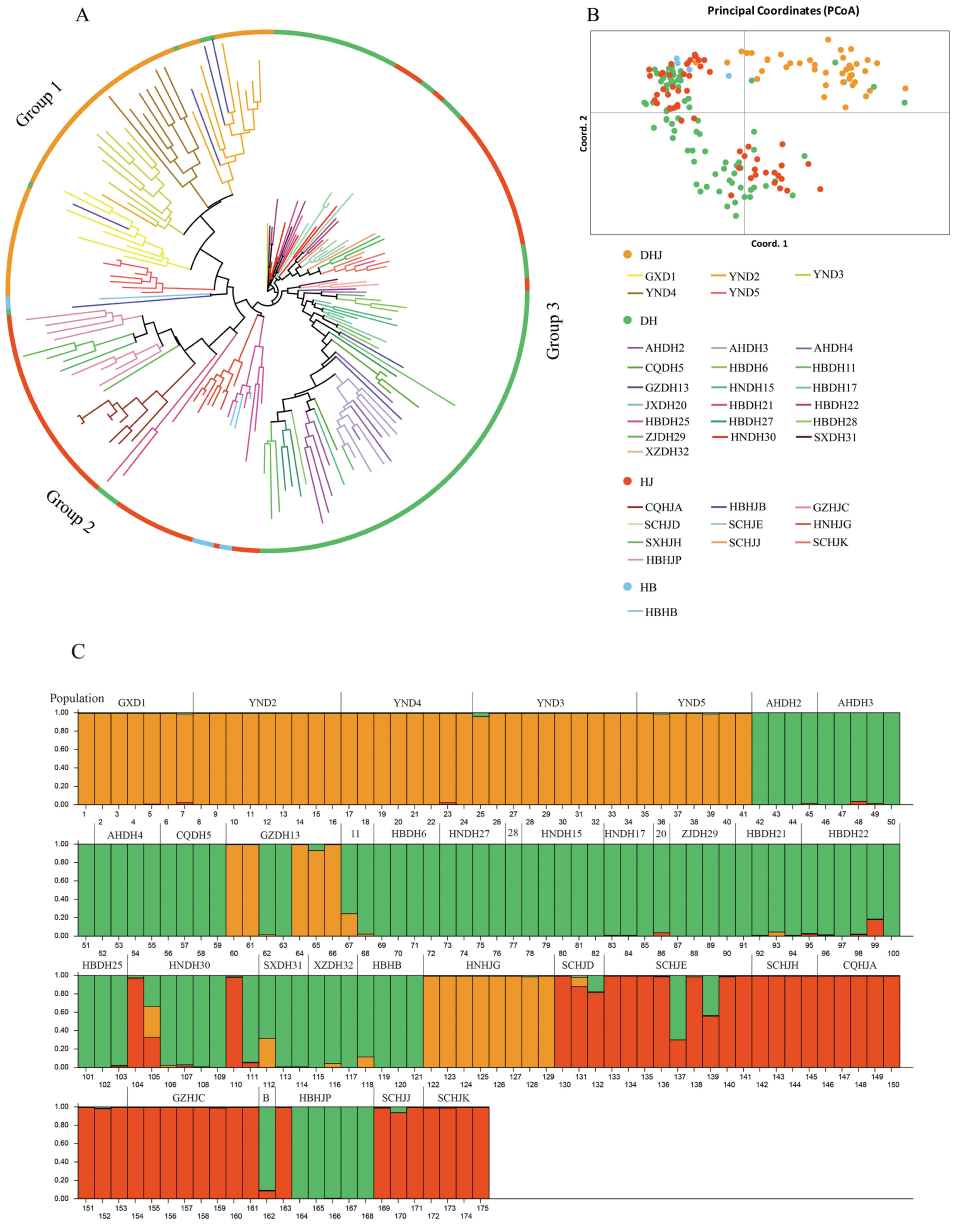
NJ tree, PCoA principal coordinates analysis and Population structure of 175 Polygonati Rhizoma germplasm. **(A)** NJ tree; **(B)** PCoA principal coordinates analysis (Germplasm from different population sources are indicated by different colored sample numbers); **(C)** Population structure of 175 Polygonati Rhizoma germplasm (K = 3) (Note: 11 is HBDH11, 28 is HBDH28, 20 is JXDH20, B is HBHJB).

The results of kinship clustering analysis of Polygonati Rhizoma populations are shown in **Figure 7A**. Group 1 included 5 P*. kingianum* (DHJ) populations, and the GZDH13 population from Qiandongnan, Guizhou Province, showed genetic component admixture with this group. This result was consistent with the lower Fst value and higher Nm value between the GZDH13 population and the *P. kingianum* (DHJ) population. Group 2 included 5 P*. sibiricum* (HJ) populations and the *P. zanlanscianense* (HB) population; notably, the HBHJB population from Xianning, Hubei Province, and the SCHJD, SCHJE, SCHJJ, and SCHJK populations from Sichuan Province all showed admixture with the *P. cyrtonema* (DH) population. Group 3 included 17 P*. cyrtonema* (DH) populations, excluding only the HBDH21 population from Shiyan, Hubei Province, and the GZDH13 population from Qiandongnan, Guizhou Province.

The results of principal component analysis (PCA) (**Figure 7B**) further verified the clustering conclusions of the neighbor-joining (NJ) tree: the *P. sibiricum* (HJ) and *P. zanlanscianense* (HB) populations had a close genetic relationship and could not be clearly divided into independent groups; in contrast, the *P. kingianum* (DHJ) population was significantly different from other groups and could be clearly clustered into an independent group.

Cluster analysis of 175 Polygonati Rhizoma germplasm samples showed that the optimal grouping was achieved when K = 3 (**Figure 7C**). The *P. kingianum* (DHJ) population had a relatively homogeneous ancestral composition, while the *P. sibiricum* (HJ), *P. zanlanscianense* (HB), and *P. cyrtonema* (DH) populations had a higher degree of genetic component admixture, indicating a closer genetic relationship among the three. Further analysis showed that most of the germplasm resources from Hubei Province were mainly assigned to the green group (corresponding to the *P. cyrtonema* (DH) population), including the HBHB, HBHJB, and HBHJP genotypes. In contrast, the genetic relationships and backgrounds of the four *P. sibiricum* (HJ) populations from Sichuan (SCHJD, SCHJE, SCHJJ, and SCHJK) were unclear; these populations showed a close genetic relationship with the *P. cyrtonema* (DH) population in kinship clustering analysis and were more closely associated with the red group of the (HJ) population in population structure analysis.

### Construction of a core collection bank

The LDSS (Linkage Disequilibrium-Stratified Sampling) strategy was used for stepwise clustering based on genetic distance, and five primary core germplasm banks were constructed sequentially (**Table 6**; **Supplementary Tables S7**-**S11**). During the clustering process, germplasm with high genetic similarity was gradually excluded, which improved the genetic diversity of the remaining samples and increased the corresponding genetic parameters: as the sampling proportion decreased, the number of alleles (*Na*) gradually decreased; in contrast, the effective number of alleles (*Ne*), Shannon's information index (I), and expected heterozygosity (*He*) all reached their maximum values when the sampling proportion was 45%. The allele retention rate of the C78 primary core germplasm bank was 84.59%, indicating that it had the lowest genetic redundancy while maintaining the most extensive genetic diversity. When the sampling proportion was lower than 45% (the proportion corresponding to C78), the reduction in germplasm materials led to significant loss of genetic variation and a decline in genetic diversity; therefore, the overall genetic diversity of the C62, C42, and C22 core germplasm banks was lower than that of C78.

**Table 6:** Genetic diversity characteristics of the original germplasm and the five primary core collections at different sampling ratios.

Collection name	Sample size	Sampling ratio	N	Na	Ne	I	Ho	He	Retention of Alleles (Ra)
Original collection	175	100%	22.264	5.139	2.817	0.997	0.016	0.465	100.00%
C103	103	59%	14.597	4.667	2.794	0.995	0.017	0.473	90.82%
C78	**78**	**45%**	**11.222**	**4.347**	**2.878**	**1.004**	**0.016**	**0.487**	**84.59%**
C62	62	35%	9.028	3.833	2.694	0.945	0.018	0.473	74.59%
C42	42	24%	6.181	3.222	2.500	0.851	0.021	0.441	62.70%
C22	22	13%	3.472	2.375	2.119	0.699	0.019	0.399	46.22%

The bold values represent the selected core collection consisting of 78 materials, as well as its genetic parameters.

**Figure 8:**
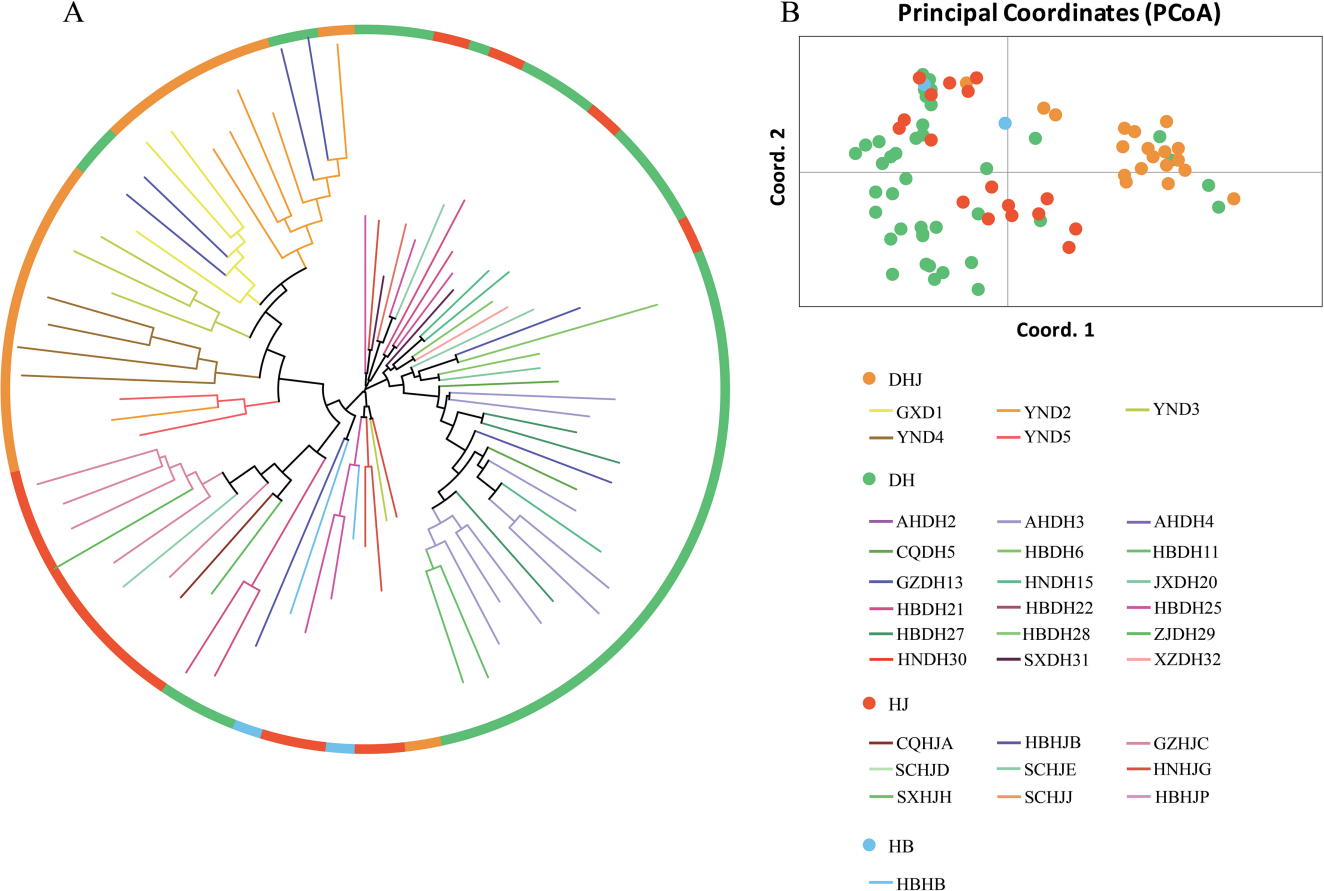
NJ tree and PCoA principal coordinates analysis of 78 Polygonati Rhizoma core collection. **(A)** NJ tree; **(B)** PCoA principal coordinates analysis. (Germplasm from different population sources are indicated by different colored sample numbers).

Cluster analysis was performed on the samples of the core germplasm bank (**Figure 8A**), and the results showed that these samples had a wide geographic distribution, covering most of the sampling areas of the original populations. The results of principal component analysis (PCA) (**Figure 8B**) indicated a high degree of consistency in the distribution pattern between the core germplasm bank samples and the original populations, suggesting that the core germplasm bank could accurately represent the genetic diversity of the original populations within the same geographic range. The above results confirm that the constructed core germplasm bank can effectively retain the genetic structure of the original populations and has high representativeness and comprehensive coverage.

## Discussion

The current identification of *Polygonatum* Mill. plant resources mainly depends on phenotypic traits. However, morphological analyses of *Polygonatum* species from different regions often exhibit similar characteristics. This similarity can lead to confusion in germplasm classification and negatively impact the conservation and development of germplasm resources.

**Figure 9:**
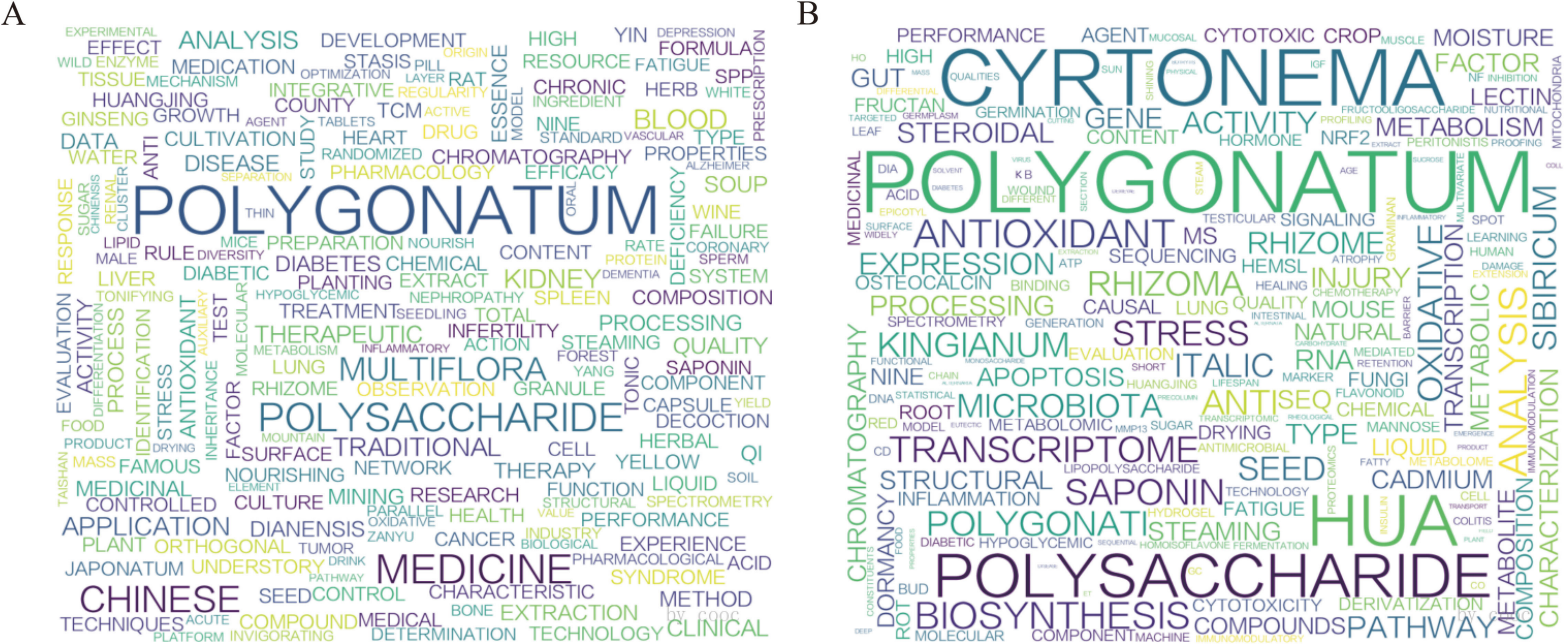
Word cloud of keywords for Polygonati Rhizoma plant research. **(A)** Chinese research keywords; **(B)** Foreign research keywords.

Simple Sequence Repeat (SSR) molecular markers, renowned for their co-dominance, high polymorphism, robust repeatability, abundance, and reliable outcomes, are pivotal tools in unraveling plant genetic diversity, species identification, breeding enhancement, and genetic map construction (Ouyang et al., 2018). A thorough investigation into genetic diversity and population structure is crucial for the conservation, exploitation, and genetic enhancement of germplasm resources. Despite the existing body of research on *Polygonatum* species that predominantly delves into their chemical composition, including polysaccharides, preparation techniques, and pharmacological activities (as shown in **Figure 9**), the deployment of SSR markers has been somewhat constrained. Although SSR markers have been established for *P. kingianum*, *P. sibiricum*, and *P. cyrtonema*, their practical application is scarce, and comprehensive studies on genetic diversity and their broader implications are infrequent (Cheng et al., 2020; Wang et al., 2018). Consequently, the present study is dedicated to a systematic exploration of the genetic diversity within *Polygonatum* species in China. Leveraging 18 highly discriminative SSR markers, we have assessed genetic diversity, kinship, and population structure across 175 medicinal *Polygonatum* accessions. Furthermore, a core collection has been meticulously curated to encapsulate the genetic diversity of these germplasm resources, thereby optimizing the number of accessions and reducing the associated management costs of germplasm banks, while simultaneously augmenting the efficient utilization of these valuable resources.

The advent of high-throughput sequencing has facilitated the incorporation of genetic data as complementary evidence for taxonomic delineation. Machine learning-based integrative approaches (e.g., BPP and DISSECT) have demonstrated superior applicability in species boundary determination, though their implementation requires extensive datasets and substantial computational resources (Karbstein et al., 2024). This study pioneers the development of two machine learning models (both achieving >81% discrimination accuracy) for *Polygonatum* species identification using SSR markers, which effectively differentiate taxa without requiring *a priori* assumptions of population genetic structure. Notably, these models enable the identification of highly informative primers, providing practical guidance for cost-efficient marker selection in future applications.

Traditional morphological classification methods for *Polygonatum* face inherent limitations due to the subjective interpretation of diagnostic characters (e.g., rhizome shape, leaf arrangement), environmental plasticity (e.g., leaf size variation under different light and soil conditions), and the frequent homoplasy of phenotypic traits (Zhu et al., 2018; Wang et al., 2020). This is consistent with our observation that some morphologically similar accessions (e.g., 3 accessions of *P. cyrtonema* from Hunan and Guizhou) showed clear genetic differentiation in the SSR-based cluster analysis (**Figure 3**), further confirming that morphological traits alone are insufficient for accurate germplasm identification—underscoring the value of our developed SSR markers for resolving taxonomic ambiguities.

The developed SSR markers exhibit exceptional interspecific discriminative power, with all 18 primer pairs showing high polymorphism (0.5 < PIC < 1.0). A comparative analysis of genetic parameters, including the number of alleles (*Na*), effective number of alleles (*Ne*), Shannon's information index (*I*), observed heterozygosity (*Ho*), and expected heterozygosity (*He*), across various Polygonati Rhizoma populations revealed that *P. cyrtonema* possessed the highest genetic diversity, followed by *P. kingianum* and *P. sibiricum*. Furthermore, *P. zanlanscianense* exhibited relatively lower genetic diversity in this study, which may be attributable to its limited sample size (n = 4). We acknowledge that further analysis with an expanded sample set will be necessary to draw definitive conclusions. Further examination of populations from different geographic regions highlighted significant genetic differentiation among populations of the same species, with pronounced differences particularly evident in *P. cyrtonema* and *P. sibiricum* populations.

A clustering analysis of 175 *Polygonatum* Mill. accessions from various regions showed that *P. kingianum* formed a distinct cluster (Group 1). In contrast, *P. sibiricum*, *P. cyrtonema*, and *P. zanlanscianense* exhibited some degree of mixing within the clustering framework. Geographically, *P. kingianum* is predominantly found in Yunnan, Sichuan, and Guizhou provinces, whereas *P. cyrtonema* has a broader distribution, including Sichuan, Guizhou, Hunan, Hubei, Henan, and Jiangxi. *P. sibiricum* is mainly distributed in Hebei, Shaanxi, Shanxi, Henan, Heilongjiang, and surrounding areas. Notably, there is overlap in the distribution areas of *P. kingianum* and *P. cyrtonema*, as well as between *P. cyrtonema* and *P. sibiricum*, which aligns with the clustering results, indicating a geographical distribution pattern that correlates with genetic variation. The Mantel test further revealed a significant positive correlation between genetic distance and geographical distance across *Polygonatum* populations, suggesting that geographic factors such as mountains, plains, water bodies, and varying climatic conditions play a major role in the genetic differentiation of populations. For instance, the GZDH13 population from Qiandongnan, Guizhou Province, exhibited a genetic background similar to that of the *P. kingianum* population, supporting this conclusion. Evidence of gene flow and introgression, both within and between populations, particularly between *P. sibiricum* populations from different regions and some *P. cyrtonema* populations, reflects the impact of long-term domestication and cultivation, which have facilitated gene flow and introgression among populations from diverse regions (Wang et al., 2020, Wang et al., 2022). In particular, certain geographically isolated populations, such as GZHJC (Guizhou) and SXHJH (Shaanxi), ZJDH29 (Zhejiang) and HBDH27 (Hubei), as well as XZDH30 (Tibet) and HBHJB (Hubei), exhibited signs of potential artificial introgression, likely due to cultivation. This was supported by their clustering patterns and genetic differentiation levels. The HBHB population, with a genetic background resembling that of the HBHJQ population from Hubei Province, may also have originated from artificial cultivation. The population structure of *P. sibiricum*, including the SCHJD, SCHJF, SCHJJ, and SCHJK populations, was more ambiguous, with genetic similarities to both *P. cyrtonema* and *P. sibiricum*. This is consistent with previous reports suggesting that the southwestern region of China may be the center of origin for *Polygonati Rhizoma* (Zhu et al., 2018).

Conserving genetic diversity within a species' entire germplasm resource is a complex and resource-intensive task. Therefore, establishing a core collection that minimizes redundancy while maximizing genetic diversity is crucial for effectively representing the species' gene pool (Anoumaa et al., 2017). In this study, we utilized the LDSS method to construct a core collection of medicinal *Polygonatum* spp. populations. To assess the representativeness of this core collection, we compared genetic parameters (allele frequency, number of alleles [*Na*], effective number of alleles [*Ne*], observed heterozygosity [*Ho*], expected heterozygosity [*He*], and Shannon's information index [*I*]) and allele retention rates between the original germplasm and the core collection. The final core collection, designated as C78, captured the genetic diversity of 33 sampled populations across 13 provinces. The representativeness of this core collection was further validated through principal component analysis (PCA), which compared the genetic structures of the core collection and the original germplasm. Additionally, the core collection geographically encompassed most of the regions from which the original germplasm was sampled.

## Conclusion

This study addresses key gaps in Polygonati Rhizoma germplasm research by leveraging 18 pairs of highly polymorphic SSR markers (average PIC unreported, but validated for strong discriminative power) to conduct comprehensive analyses of population genetics, phylogenetic relationships, and population structure across 175 *Polygonatum* accessions—yielding three novel, actionable findings:

First, our SSR-based analysis delineated three distinct genetic groups with clear taxonomic and geographic relevance: Group 1 (exclusively *P. kingianum*), Group 2 (*P. sibiricum* + *P. zanlanscianense*), and Group 3 (*P. cyrtonema* + Sichuan-sourced *P. sibiricum*). This resolution resolves prior ambiguity in *Polygonatum* intraspecific classification, providing a genetic framework for accurate germplasm identification—critical for avoiding mismanagement of conservation resources.

Second, we established a core germplasm collection, with the C78 primary core collection standing out for its minimal genetic redundancy while effectively capturing the geographic origin and full genetic diversity of the original accessions. This is the first systematic Polygonati Rhizoma core collection built via SSR-guided screening, offering a cost-efficient solution to address the resource-intensive challenge of conserving large germplasm pools.

These results deliver dual value for Polygonati Rhizoma germplasm conservation: The genetic grouping clarifies priority populations (e.g., geographically distinct *P. kingianum* in Group 1, genetically unique Sichuan *P. sibiricum* in Group 3) for targeted *in-situ* protection, while the C78 collection enables streamlined *ex-situ* conservation and germplasm regeneration. Additionally, the 18 SSR markers and genetic grouping data provide technical support for future genetic improvement—facilitating selection of diverse parental materials (e.g., crossbreeding between Group 1 and Group 3 accessions) to develop high-quality *Polygonatum* varieties.

Future work will expand the *P. zanlanscianens*e sample size to refine its phylogenetic placement in Group 2, and combine SSR data with medicinal component traits to accelerate marker-assisted breeding—ultimately advancing sustainable utilization of Polygonati Rhizoma germplasm resources.

## Data Availability

The datasets presented in this study can be found in online repositories. The names of the repository/repositories and accession number(s) can be found in the article/**Supplementary Material**.
