# Supplementary figures and images for: Genetic diversity and core collection of Polygonati Rhizoma in China via SSR markers
Source: Front Plant Sci. 2025 Nov 6;16:1674396. doi: 10.3389/fpls.2025.1674396 (PMC12631632; doi:10.3389/fpls.2025.1674396)

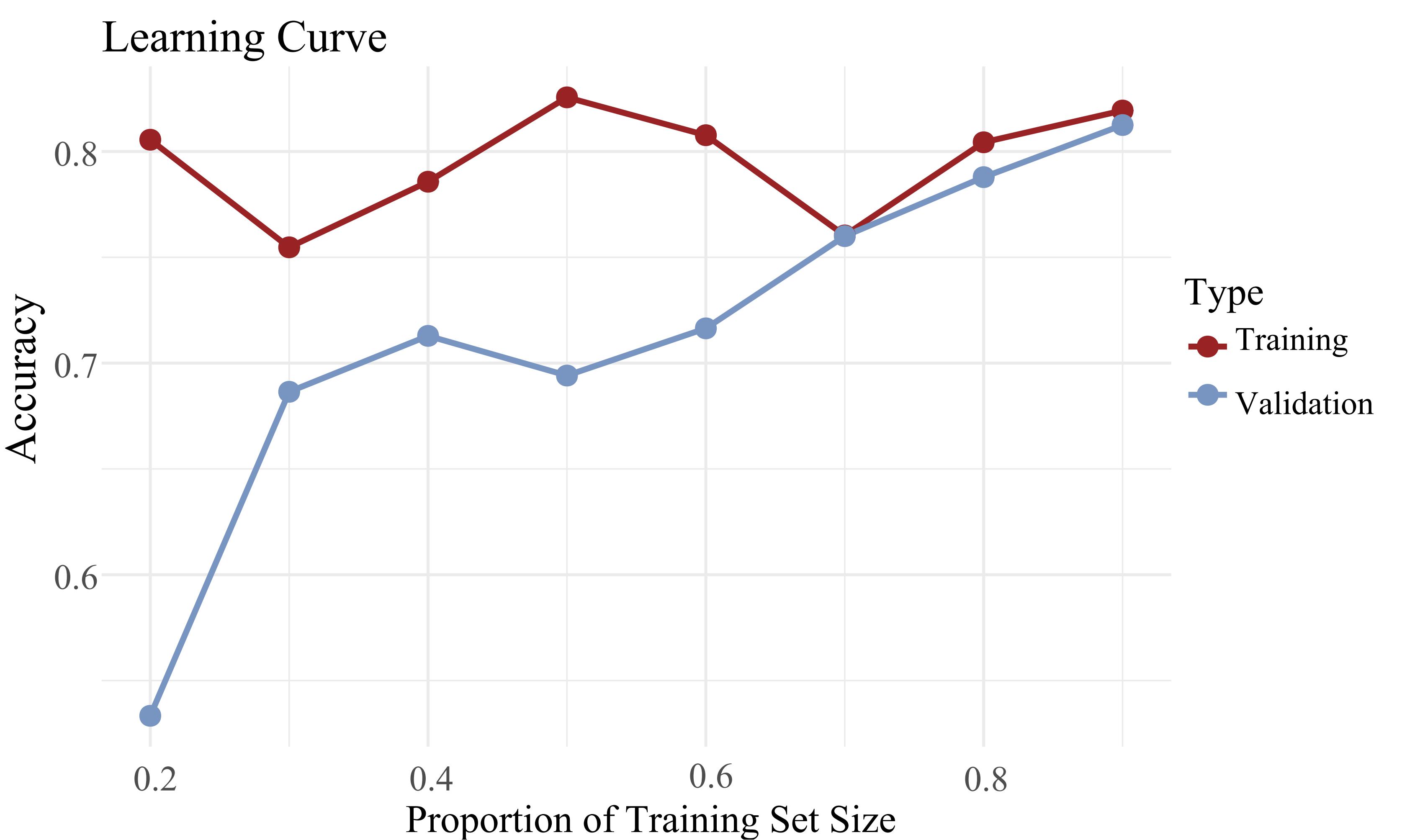

Supplement: Supplementary file 2 [file Image2.jpeg]
